# The immunoregulatory role of p21 in the development of the temporomandibular joint‐osteoarthritis

**DOI:** 10.1002/cre2.404

**Published:** 2021-02-10

**Authors:** Tsendsuren Khurel‐Ochir, Takashi Izawa, Akihiko Iwasa, Fumiya Kano, Akihito Yamamoto, Eiji Tanaka

**Affiliations:** ^1^ Department of Orthodontics and Dentofacial Orthopedics Tokushima University Graduate School of Oral Sciences Tokushima Japan; ^2^ Department of Orthodontics Okayama University Graduate School of Medicine, Dentistry, and Pharmaceutical Sciences Okayama Japan; ^3^ Department of Orthodontics and Dentofacial Orthopedics Institute of Biomedical Sciences, Tokushima University Graduate School Tokushima Japan; ^4^ Department of Tissue Regeneration Institute of Biomedical Sciences, Tokushima University Graduate School Tokushima Japan

**Keywords:** chondrocyte, metalloprotease, osteoarthritis, p21, temporomandibular joint

## Abstract

**Objective:**

We aimed to identify the immunoregulatory role of the cyclin‐dependent kinase inhibitor p21 in the homeostasis of mandibular condylar cartilage affected by mechanical stress.

**Materials and methods:**

Ten C57BL/6 wild‐type (WT) and ten p21^−/−^ mice aged 8 weeks were divided into the untreated and treated groups. In the treated groups, mechanical stress was applied to the temporomandibular joint (TMJ) through forced mouth opening for 3 hr/day for 7 days. The dissected TMJs were assessed using micro‐CT, histology, and immunohistochemistry.

**Results:**

Treated p21^−/−^ condyles with mechanical stress revealed more severe subchondral bone destruction, with thinner cartilage layers and smaller proteoglycan area relative to treated WT condyles; untreated WT and p21^−/−^ condyles had smoother surfaces. Immunohistochemistry revealed significant increases in the numbers of caspase‐3, interleukin‐1*β*, matrix metalloprotease (MMP)‐9, and MMP‐13 positive cells, and few aggrecan positive cells, in treated p21^−/−^ than in treated WT samples. Moreover, the number of TUNEL positive cells markedly increased in p21^−/−^ condyles affected by mechanical stress.

**Conclusions:**

Our findings indicate that p21 in chondrocytes contributes to regulate matrix synthesis via the control ofm aggrecan and MMP‐13 expression under mechanical stress. Thus, p21 might regulate the pathogenesis of osteoarthritis in the TMJ.

## 1 INTRODUCTION

Osteoarthritis (OA), the most common degenerative joint disease, has affected adults worldwide (Sharma, Kapoor, & Issa, 2006; Zhang & Jordan, 2010). Its main symptoms are joint pain and swelling, stiffness, and subsequent motion disability. Prevalence of symptomatic OA increases with age, and in particular, more than 75% of the population aged over 65 years are reported to be affected (Brooks, 2006); however, preventive therapies for OA onset and progression are still ongoing (Zhang & Jordan, 2010).

Like knee and hip OA, osteoarthritis in the temporomandibular joint (TMJ‐OA) is characterized by cartilage destruction with surface roughness, and abnormal remodeling of the subchondral bone (Tanaka, Detamore, & Mercuri, 2008). During progression, degenerative microenvironments of TMJ‐OA promote cartilage matrix destruction, apoptosis and necrosis of chondrocytes, and abnormal subchondral bone resorption (Kuroda et al., 2009; Wang, Zhang, Gan, & Zhou, 2015), resulting in irreversible articular collapse and functional impairment. Because of the limited self‐repairing ability of the mandibular condylar cartilage, further investigations are needed to exploit effective treatment remedy to prevent the onset and progression of TMJ‐OA.

The major cause of mandibular condylar cartilage breakdown, as for that of knee cartilage, is overloading (Nitzan, 2001; Tanaka et al., 2008). Chondrocytes, and especially hypertrophic chondrocytes, are assumed to have enhanced mechanoresponsive mechanisms (Wong, Siegrist, & Goodwin, 2003). Excessive mechanical stress may alter the metabolism of mandibular condylar chondrocyte and decrease the amount of extracellular matrix (Fujita et al., 2019; Kuroda et al., 2009; Ogasawara et al., 2020). In articular cartilage affected by OA, reduction of the amounts of extracellular matrix such as type II collagen and aggrecan (ACAN) is caused by the decrease in protein synthesis from chondrocytes, and the activation of extracellular matrix degrading enzymes including matrix metalloproteinases (MMPs) and a disintegrin and MMP with thrombospondin motifs, which are major cartilage aggrecanases in humans (Curtin & Reville, 1995; Sakata et al., 2020). The reduction of ACAN through aggrecanase enzyme activity is a key step in early OA (Huang & Wu, 2008). MMPs, and especially MMP‐13, are fatal enzymes associated with the degradation of articular cartilage in OA (Sakata et al., 2020; Sekino et al., 2018).

Initially, the cyclin‐dependent kinase inhibitor, p21 has been identified as a potent inhibitor of cell cycle progression (el‐Deiry et al., 1993; Gu, Kuntz‐Simon, Rommelaere, & Cornelis, 1999 and Xiong et al., 1993). p21 is likely to mediate cell proliferation and inflammation after injury (Olive et al., 2008), and plays important roles in the subsequent cytostasis and cell death (Suzuki, Suzuki, Tsutomi, Akahane, Araki, & Miura, 1998). A recent report indicates that p21 deficiency shows catabolic effects via the moderation of ACAN and MMP‐13 expression through signal transducer and activator of transcription 3 (STAT3) phosphorylation in knee cartilage (Hayashi et al., 2015). Furthermore, p21‐deficient mice exhibit enhanced joint inflammation after mechanical stimulation, which leads to OA susceptibility (Kihara et al., 2017). However, little information on the immunoregulatory role of p21 in the onset and progression of TMJ‐OA is available.

Thus, we hypothesized that p21 deficiency regulates extracellular matrix synthesis in response to mechanical stress applied to mandibular condylar cartilage. This study was conducted to identify the function of p21 in TMJ cartilage homeostasis using p21 knockout (p21^−/−^) mice.

## 2 MATERIALS AND METHODS

### 2.1 Mice

Ten C57BL/6 wild‐type (WT) and ten p21^−/−^ mice aged 8 weeks were used in this study. Homozygous B5.12956 (Cg)‐Cdkn1a^tm1Led^/J mice were obtained from the Jackson Laboratory (Bar Harbor, ME, USA) as p21^−/−^ mice. These mice were back‐bred against a C57BL/6 background. All mice used in this study were backcrossed for less than 10 generations, and p21^+/+^ littermates were used as WT mice. Genotyping was executed by polymerase chain reaction amplification of murine tail DNA using allele‐specific probes.

### 2.2 Mouse model of mechanical stress‐induced TMJ‐OA

Each mouse was kept separately in plastic cages maintained at ambient temperature (22–24°C) with a 12‐hr light/dark cycle. They were fed a solid diet and had free access to water throughout the experimental period. The p21^−/−^ and WT mice each were divided randomly into untreated and treated groups (*n* = 5/group): no mechanical stress (untreated) and mechanical stress (treated).

In the treated groups, the mandibular condyle received mechanical stress via forced mouth opening for 3 hr/day for 7 days by using a custom‐made device. The device was used to keep the mandible in a maximal open‐mouth position (14 mm) and deliver approximately 2 N force to each TMJ (Izawa et al., 2016). In the mouth‐opening procedure, the mice were anesthetized with an intraperitoneal injection of 50 mg/kg somnopentyl. The same anesthesia schedule was used in the untreated groups, with no mechanical stress applied (Figure S1).

After the whole experiments, all mice were sacrificed and the TMJs tissue blocks were dissected for histological, immunohistochemical, and micro‐computed tomography (μCT) analyses. All procedures executed in this study were permitted by the Tokushima University Animal Care and Use Committee (permit no. T2019‐70) and carried out according to the ARRIVE guidelines (Figure S2).

### 2.3 Micro‐computed tomography (μCT) analysis

The condyles resected from murine mandibles were separated carefully from the surrounding tissues and fixed in 70% ethanol overnight. The dissected condyles were then examined in high‐resolution μCT (SkyScan 1176 scanner and analytical software; Buruker, Billerica, MA). Series images were acquired at 50 kV and 200 μA. During image acquisition, the condyles were tightly wrapped with a plastic sheet to restrict movement and dehydration. Then, thresholding was established to distinguish the bone image from the background. Each μCT image had a resolution of 9 μm/pixel. The posterior region of the mandibular condyle on the midsagittal section was defined as the region of interest, in which microstructural parameters, including the ratio of bone volume, trabecular thickness, and trabecular separation, were evaluated.

### 2.4 Tissue preparation and histological staining

The tissue blocks of the TMJ were fixed with 4% paraformaldehyde prepared with ethylenediaminetetraacetic acid in phosphate‐buffered saline (PBS) for 20 consecutive days. With a microtome (HM360; Carl Zeiss Jena, Germany), serial sagittal sections were cut from the paraffin‐embedded tissue blocks. For each condyle, the section was stained with hematoxylin and eosin (HE) for histological evaluation, and counterstained with 0.02% Fast Green and 0.1% Safranin‐O and Toluidine blue. Safranin‐O and Toluidine blue are useful dyes to detect the extracellular matrix of cartilage; however, they recognize different components of glycoproteins. Safranin‐O mainly stains proteoglycan, such as aggrecan and decorin, while Toluidine blue stains mainly glycosamine glycan, such as hyaluronan, chondroitin sulfate, and keratan sulfate: thereby, the staining patterns of them were different. Tartrate‐resistant acid phosphatase (TRAP; 387‐A; Sigma, St. Louis, MO) activity was also examined to detect active bone‐resorbing osteoclasts according to the manufacturer's instructions.

### 2.5 Immunohistochemistry

To elucidate the expression of ACAN (1:200; ab36861, Abcam, Cambridge, United Kingdom), MMP‐9 (1:1000; ab38898, Abcam), MMP‐13 (1:1000; ab39012, Abcam), interleukin (IL)‐1β (1 μg/ml; ab9722, Abcam), and cleaved caspase‐3 (1:100; 9509S, Cell Signaling Technology, Danvers, MA), and apoptosis marker in the mandibular condylar cartilage, immunohistochemical staining was carried out using several primary antibodies (Immuno Biological Laboratories, Fujioka, Japan). After deparaffinization and blocking, the sections were incubated overnight with primary antibodies diluted in phosphate‐buffered saline/0.1% bovine serum albumin 4°C in a humid atmosphere. Immunostaining was performed using a Histofine simple stain kit (Nichirei, Tokyo, Japan). Briefly, after blocking of endogenous peroxidase activity with 0.3% hydrogen peroxide in methanol, non‐specific binding of the antibody was blocked by incubating the section for 30 min with non‐specific staining blocking reagent (Dako, Carpinteria, CA). After washing with PBS, the sections were incubated with the corresponding secondary antibodies for 1 hr at room temperature. Finally, immunoactivity was detected using diaminobenzidine, followed by counter‐staining with Mayer's hematoxylin. The sections were observed under a BioRevoBZ‐9000 microscope (KEYENCE, Osaka, Japan).

### 2.6 TUNEL staining

The apoptotic chondrocytes were detected by the TdT‐mediated dUTP‐digoxigenin nick‐end labelling (TUNEL) method. This can label specifically the 30‐hydroxyl termini of DNA strand breaks. TUNEL staining was carried out using an apoptosis in situ detection kit (Wako Pure Chemical, Osaka, Japan) according to the manufacturer's instructions, and stained apoptotic cells were then classified via microscopy (KEYENCE).

### 2.7 Histometric analysis

A representative sagittal section was selected from the midsagittal plane of the condyle in each animal. For each section, the mandibular condylar cartilage was divided into three regions: anterior, intermediate, and posterior regions. In the posterior region, where the damage had been induced via mouth opening, the numbers of chondrocytes showing positivity for ACAN (1:200; ab36861, Abcam, Cambridge, United Kingdom), MMP‐9 (1:200; ab38898, Abcam), MMP‐13 (1:200; ab39012, Abcam), IL‐1β (1 μg/ml; ab9722, Abcam), and cleaved caspase‐3 (1:100; 9509S, Cell Signaling Technology, Danvers, MA) were counted within a fixed measuring frame (430 × 1470 μm). In one TRAP‐stained section through the midsagittal plane of the condyle per animal, the number of osteoclasts in the mineralized layer subjacent to the hypertrophic cell layer was counted. TRAP‐positive cells with two or more nuclei were counted as active osteoclasts. The modified Mankin scoring system (Xu et al., 2003) was used to elucidate the score of cartilage degeneration in the mandibular condyle. Scoring was based on pericellular and background Safranin‐O staining, chondrocyte arrangement, and cartilage structural feature. Score 0 indicates healthy cartilage, with higher scores indicating higher degeneration up to a maximum score of 6. The sections were analyzed by three independent experts who were blinded to the type of the samples analyzed.

### 2.8 Statistical analysis

The experiments were repeated in at least triplicate for each set of conditions, and reproducibility of these results was confirmed by at least two sets of experiments. Means and *SDs* were calculated from the data obtained. Statistical analysis was performed *t* test or one‐way ANOVA followed by Tukey honest significant differences test as a post hoc test to examine mean differences at the 5% level of significance. Mankin scores were analyzed using the Wilcoxon signed rank‐sum test carried out with the statistical package R (version 4.0.2; available as a free download from https://www.r-project.org/).

## 3 RESULTS

### 3.1 μCT properties

**FIGURE 1:**
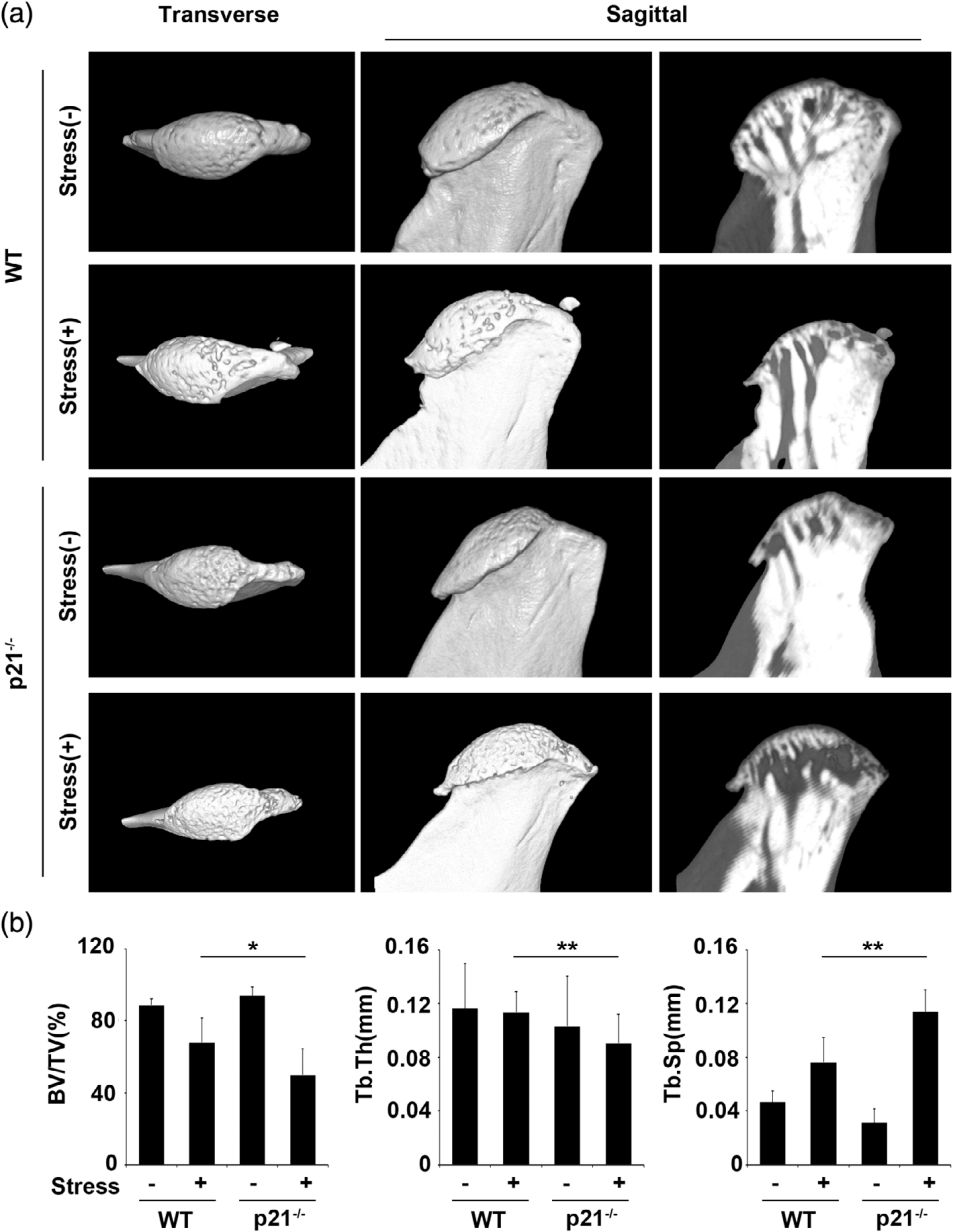
Effects of p21 deficiency on murine mandibular condyles. (a) Three‐dimensional reconstructions of μCT images from 8‐week‐old wild‐type and p21^−/−^ mandibular condyles with treated and untreated groups, with representative transverse and sagittal sections. (b) Trabecular bone volume (BV) was determined in representative sagittal sections, and the bone fraction volume was presented as the BV/TV ratio. Tb.Sp and Tb.Th indicate trabecular separation and thickness, respectively. The data presented are the means ± *SDs* (*n* = 5/group). * *p* < .05, ** p < .01; BV, bone volume; (TV), Tb.Sp, trabecular separation; Tb.Th, trabecular tickness; TV, tissue volume

The μCT images showed that the condylar cartilage surfaces were rougher, with severe subchondral bone defects, in the treated groups with mechanical stress, and smoother with few or no subchondral bone defects in the untreated groups (Figure 1a). Relative to WT condyles subjected to mechanical stress, p21^−/−^ condyles subjected to such stress displayed more severe surface roughness with small concavities. The application of mechanical stress to the mandibular condyles decreased the ratio of bone volume and trabecular thickness, and these values were significantly lower in p21^−/−^ condyles subjected to mechanical stress than in their WT counterparts (Figure 1b; both *p* < .01). In contrast, the trabecular separation was significantly greater in p21^−/−^ than in WT condyles subjected to mechanical stress (Figure 1b; *p* < .01).

### 3.2 Histological properties

**FIGURE 2:**
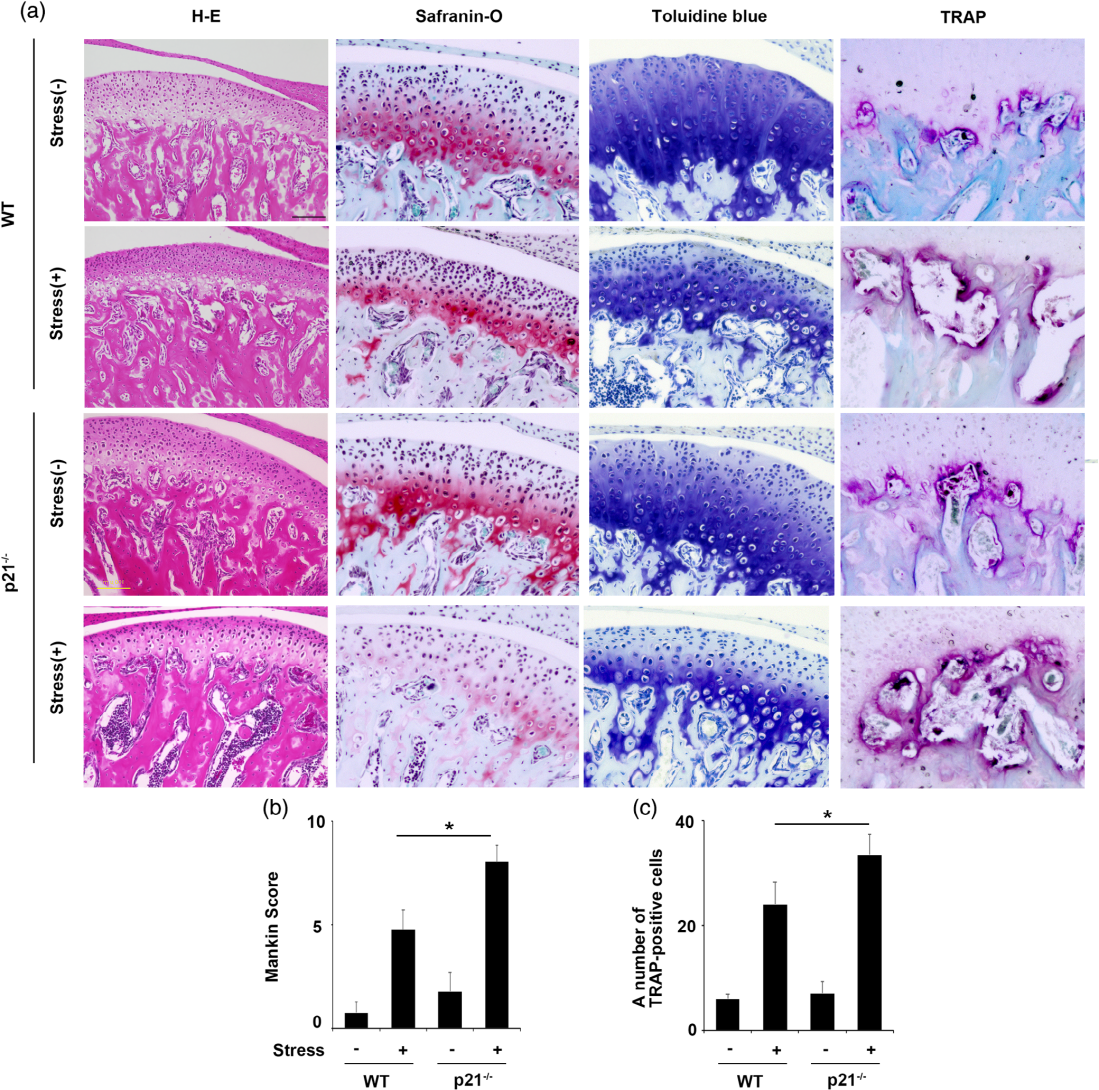
Effects of p21 deficiency on murine mandibular condylar cartilage and subchondral bone. Histological findings of the mandibular condylar cartilage obtained from treated and untreated groups of 8‐weeks‐old wild‐type (WT) and p21^−/−^ mice were observed following the staining of tissue sections with hematoxylin and eosin (HE), safranin‐O/fast green, toluidine blue (a) and tartrate‐resistant acid phosphatase (TRAP) (b). (c) Histological grading (modified Mankin scores) of mandibular condylar cartilage obtained from treated and untreated groups of WT and p21^−/−^ mice. The data are presented are means ± *SDs* (*n* = 5/group). (d) Numbers of TRAP‐positive cells in subchondral bone obtained from the treated and untreated WT and p21^−/−^ mice. Data have presented the means ± *SDs* (*n* = 5/group). Scale bar = 100 μm. * *p* < .05

OA‐like lesions, characterized by decreased cartilage thickness, increased local cell‐free area, irregular alignment of chondrocytes, cartilage surface roughness, and fibrillation of the superficial layer, were observed in all condylar cartilage subjected to mechanical stress (Figure 2a). Furthermore, mandibular condylar cartilage from WT mice subjected to mechanical stress had significantly lower modified Mankin score than p21^−/−^ mice subjected to the same stress. (Figure 2b; *p* < .05). TRAP‐positive osteoclasts were detected in the mineralized subchondral bone layers of WT and p21^−/−^ condyles (Figure 2a). The number of TRAP‐positive cells was significantly greater in p21^−/−^ than in WT condyles subjected to mechanical stress (Figure 2c; *p* < .05).

### 3.3 Expression of ACAN, MMP‐9, MMP‐13, and IL‐1β in articular cartilage

**FIGURE 3:**
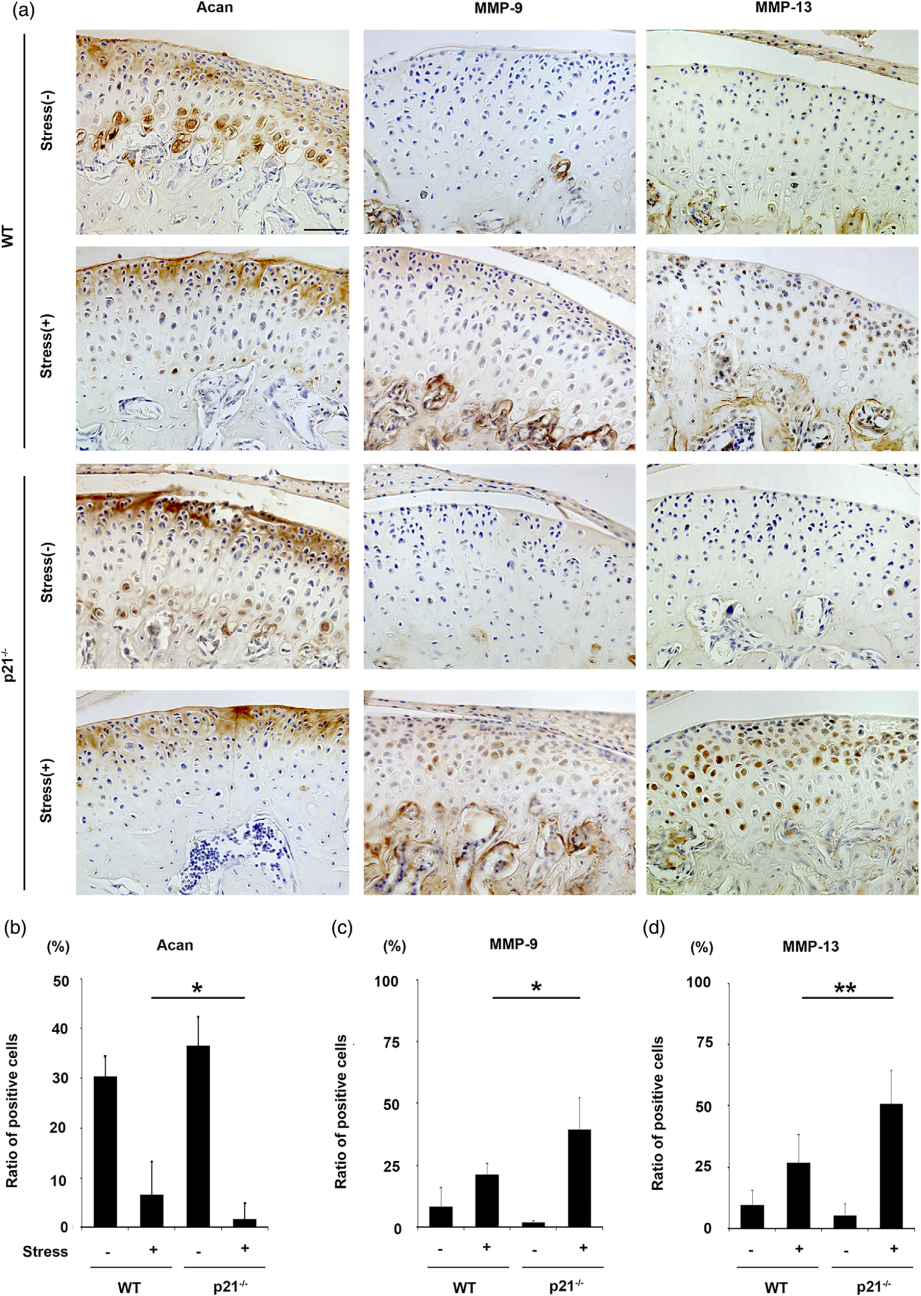
Immunohistochemical staining for aggrecan (ACAN), matrix metalloproteinase (MMP)‐9 and MMP‐13 in mandibular condylar cartilage obtained from treated and untreated groups of 8‐week‐old wild‐type (WT) and p21^−/−^ mice (a). Numbers of ACAN, MMP‐9 and MMP‐13‐positive cells in mandibular condylar cartilage of treated and untreated groups of WT and p21^−/−^ mice (b, c, d). The data presented are the means ± *SDs* (*n* = 5/group). Scale bar = 100 μm. * *p* < .05, ** *p* < .01

**FIGURE 4:**
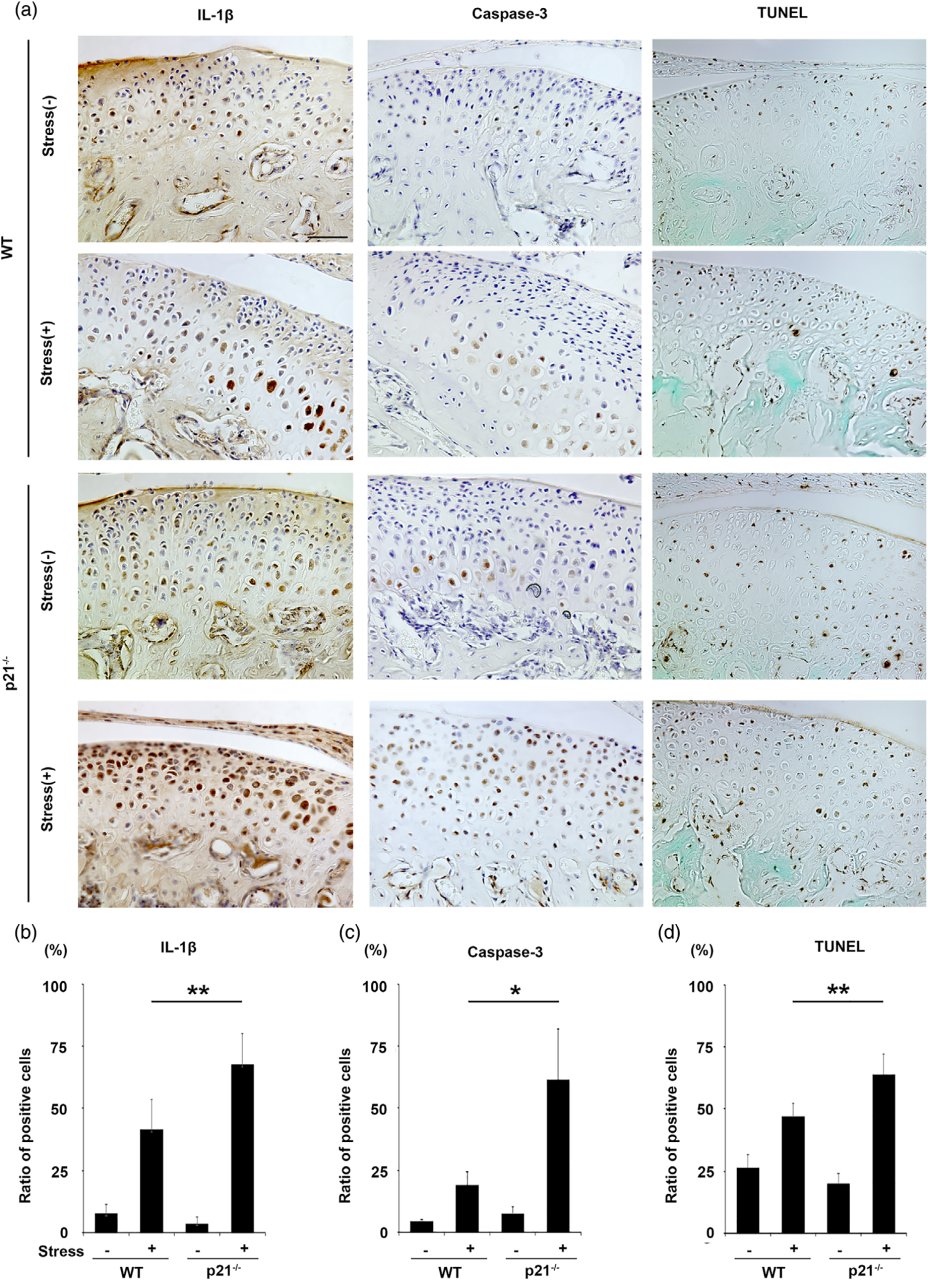
Immunohistochemical analysis of phosphorylated interleukin (IL)‐1β and cleaved caspase‐3, and TUNEL staining of murine mandibular condylar cartilage obtained from treated and untreated groups of 8‐week‐old wild‐type (WT) and p21^−/−^ mice (a). Numbers of IL‐1β, cleaved caspase‐3, and TUNEL‐positive cells in mandibular condylar cartilage of treated and untreated groups of WT and p21^−/−^ mice (b, c, d). The data presented are the means ± *SDs* (*n* = 5/group). Scale bar = 100 μm. * *p* < .05, ** *p* < .01

ACAN expression was significantly lesser in p21^−/−^ than in WT cartilage subjected to mechanical stress (Figure 3a, b; *p* < .05). The expression of MMP‐9 and MMP‐13 was increased in both treated groups with mechanical stress relative to the untreated groups (Figure 3a), with significantly higher levels observed in p21^−/−^ subjected to mechanical stress (Figure 3a, c, d; *p* < .05; *p* < .01). Significantly more IL‐1β–positive chondrocytes were observed in p21^−/−^ than in WT cartilage subjected to mechanical stress (Figure 4a, b; *p* < .01).

### 3.4 Chondrocyte apoptosis in the subchondral condylar bone

The number of chondrocytes showing immunopositivity for cleaved caspase‐3, an apoptosis marker, was higher in p21^−/−^ relative to WT cartilage subjected to mechanical stress (Figure 4a, c; *p* < .01). Meanwhile, the number of TUNEL‐positive cells in p21^−/−^ cartilage subjected to mechanical stress was significantly higher than that in WT cartilage subjected to mechanical stress (Figure 4a, d; *p* < .05).

## 4 DISCUSSION

Different from rheumatoid arthritis, TMJ‐OA is a primarily non‐inflammatory disease and one of its most common causes is mechanical overloading (Arnett, Milam, & Gottesman, 1996; Tanaka et al., 2008). Thus, the secondary inflammation following the mechanical overloading to mandibular condyle plays a crucial role in TMJ‐OA onset and progression. In early OA, a transient upregulation of chondrocyte proliferation is involved in the enhancement of cartilage matrix protein synthesis. Chondrocytes appear to have enhanced mechanoresponsive mechanisms (Wong et al., 2003). The end‐stage of OA includes a collapse of articular cartilage, particular extracellular matrix destruction by MMPs released from inflammatory chondrocytes (Kuroda et al., 2009; Sakata et al., 2020; Shlopov et al., 1997). In previous work (Ogasawara et al., 2020), we developed the mouse model of mechanical stress‐induced TMJ‐OA that was used in the present study. It has been reported that mechanical stress induced by forced mouth opening decreases the subchondral bone volume in mice (Ogasawara et al., 2020; Sobue et al., 2011). In rabbit and rat models, repetitive steady jaw opening successfully created OA‐like changes compatible with the clinical signs for human TMJ‐OA (Fujisawa et al., 2003; Kawai et al., 2008). Our results support previous findings that mechanical overloading of the TMJ induced an increase in MMP‐13–positive chondrocytes and a decrease in ACAN synthesis, leading to TMJ‐OA‐like lesions in p21^+/+^ and p21^−/−^ condyles. These results imply that our protocol of daily forced mouth opening is useful for the creation of an experimental TMJ‐OA model for the evaluation of the onset and progression of this condition.

Although p21 is a potent inhibitor of cell cycle progression, there were no obvious differences in body weight, size, and health between p21^−/−^ and WT mice. The tooth number, size, and shape of p21^−/−^ were normal. Hayashi et al. (2015) investigated the role of p21 in cartilage homeostasis, focusing on the relationship between p21 and STAT3, in human chondrocytes derived from the knee joint. They reported that p21 deficiency results in catabolic effects via the mediation of ACAN and MMP‐13 expression through STAT3 phosphorylation in knee cartilage. Kihara et al. (2017) examined joint inflammation to assess the mechanisms related to p21 function in OA progression using p21‐knockout mice and concluded that p21‐deficient mice with surgery‐induced destabilization of the medial meniscus were susceptible to alteration of the OA phenotype in the knee, which is characterized by upregulated osteoclast expression, macrophage infiltration, and MMP‐13 expression via IL‐1β‐induced nuclear factor (NF)‐kB signaling. Although many reports have described the function of p21 in articular cartilage, few reports describe its role in TMJ‐OA, despite the frequent occurrence of this disease. To the best of our knowledge, the present study is the first to examine the role of p21 deficiency in the mandibular condylar cartilage in the context of TMJ‐OA.

We observed increased numbers of chondrocytes undergoing cell death in the TMJ after mechanical stress in p21^−/−^ and WT condyles. Chondrocytes that did not die revealed alterations of extracellular matrix synthesis or degradation of this matrix. In addition, we observed severe cartilage destruction related to greater inflammation in p21‐deficient mice in this model of mechanical stress‐induced TMJ‐OA. p21 deficiency was found to promote cartilage degeneration, increase the numbers of apoptotic cells, and TRAP‐positive activated osteoclasts, increase the expression of MMP‐13 and IL‐1β, and decrease ACAN synthesis in TMJ‐OA cartilage. Apoptosis is an evolutionarily conserved programme that leads to cell death, which plays roles in normal development and the maintenance of homeostasis in adults. As p21 is a potent inhibitor of cell cycle progression, p21 deficiency might affect the chondrocyte cycle under overloading, resulting in the promotion of cell death.

Regarding the mechanism involved in secondary inflammation in p21^−/−^ mice with TMJ‐OA‐like lesions, IL‐1β is known to stimulate NF‐kB signaling and induce the expression of MMP‐3 and MMP‐13 (Mengshol, Vincenti, Coon, Barchowsky, & Brinckerhoff, 2000), and the IKK complex plays a crucial role in controlling NF‐kB activity (Ghosh & Karin, 2002). In this study, we confirmed that MMP‐9, MMP‐13, and IL‐1β expression levels were upregulated in the mechanically stressed mandibular condylar cartilage of p21‐deficient mice. These findings suggest that the lack of p21 enhances MMP‐13 expression in mandibular condylar cartilage via IL‐1β‐induced NF‐kB signaling activation. We also indicated that p21 deficiency altered osteoclastogenesis in the subchondral mandibular bone. Few previous studies have reported the regulatory role of p21‐activated kinases (PAKs) in OA; they demonstrated that IPA‐3, a PAK inhibitor, reduced cartilage degradation associated with OA and that the suppression of PAK2 promoted R‐Smad activation in the transforming growth factor (TGF)/Smad signaling pathway in chondrocytes (Hu et al., 2019). Using Smad3 knockout mice, we previously examined the molecular mechanisms by which crosstalk between TGF‐β/Smad3 and sphingosine 1‐phosphate (S1P)/S1P receptor signaling may occur to maintain the condylar cartilage and subchondral bone and prevent TMJ‐OA (Mori, Izawa, & Tanaka, 2015). We demonstrated that chondrocyte maintenance, which involves cell motility and apoptosis, is mediated by such crosstalk. Together with those findings, the present results indicate that p21 deficiency affects cartilage and subchondral bone homeostasis via a breakdown of crosstalk between the TGF‐β/Smad3 and S1P/S1P3 signaling pathways. Activation of the NF‐κB pathway is a key step in RANKL‐induced osteoclast differentiation, and it occurs following the targeting of IκBα (Inhibitor kappa B‐alpha) for ubiquitin‐dependent degradation (Takayanagi, 2007). Thus, p21 appears to be able to target NF‐kB and TGF‐β/Smad signaling, which negatively affects the formation of osteoclasts from macrophages stimulated by RANKL, as well as chondrocyte and osteoclast differentiation.

## 5 CONCLUSION

In conclusion, p21 in chondrocytes might play a crucial role to regulate extracellular matrix synthesis via the control of ACAN and MMP‐13 expression under mechanical stress. This cell cycle‐related molecule may regulate TMJ‐OA pathogenesis in mice through IL‐1β‐induced NF‐kB signaling activation. The TMJ exhibits unique characteristics that easily distinguish it from other synovial joints in humans. Correspondingly, TMJ disorders, symptoms, and disease distribution are different from those of other joint disorders. As TMJ‐OA‐specific treatments may be required, continued efforts to better understand the pathogenesis of TMJ‐OA are needed to exploit effective new therapies.

## AUTHOR CONTRIBUTIONS

Study design: Tsendsuren Khurel‐Ochir, Takashi Izawa, Akihito Yamamoto, and Eiji Tanaka.

Data acquisition: Tsendsuren Khurel‐Ochir, Akihiko Iwasa, Fumiya Kano, and Akihito Yamamoto.

Data analysis: All authors.

Writing of first draft: Tsendsuren Khurel‐Ochir, Eiji Tanaka, and Akihito Yamamoto.

Manuscript revision and approval of final manuscript: All authors.

## Supporting information


**Figure S1.** The experiment and were randomly divided into two untreated and two treated groups: p21^−/−^ and WT without mechanical stress for control groups; p21^−/−^ and WT with mechanical stress for experimental groups (*n* = 5 for each group). In the treated groups, mechanical stress was applied to the TMJs by forced mouth opening for 3 hr/day for 7 days. This device kept the mandible in a maximal mouth opening position of 14 mm and delivered a force of 2 N on each TMJ

**Figure S2.** Overview of experimental design (white box) and workflow (grey box)

## Data Availability

The data that supports the findings of this study are available in the supplementary material of this article. In addition, the data that support the findings of this study are available from the corresponding author upon reasonable request.
