# Supplementary material for: The immunoregulatory role of p21 in the development of the temporomandibular joint‐osteoarthritis
Source: Clin Exp Dent Res. 2021 Feb 10;7(3):313–22. doi: 10.1002/cre2.404 (PMC8204032; doi:10.1002/cre2.404)
Supplement: Supplementary file 1 — Figure S1. The experiment and were randomly divided into two untreated and two treated groups: p21−/− and WT without mechanical stress for control groups; p21−/− and WT with mechanical stress for experimental groups (n = 5 for each group). In the treated groups, mechanical stress was applied to the TMJs by forced mouth opening for 3 hr/day for 7 days. This device kept the mandible in a maximal mouth opening position of 14 mm and delivered a force of 2 N on each TMJ [file CRE2-7-313-s001.pdf]

Supplemental Figure 1

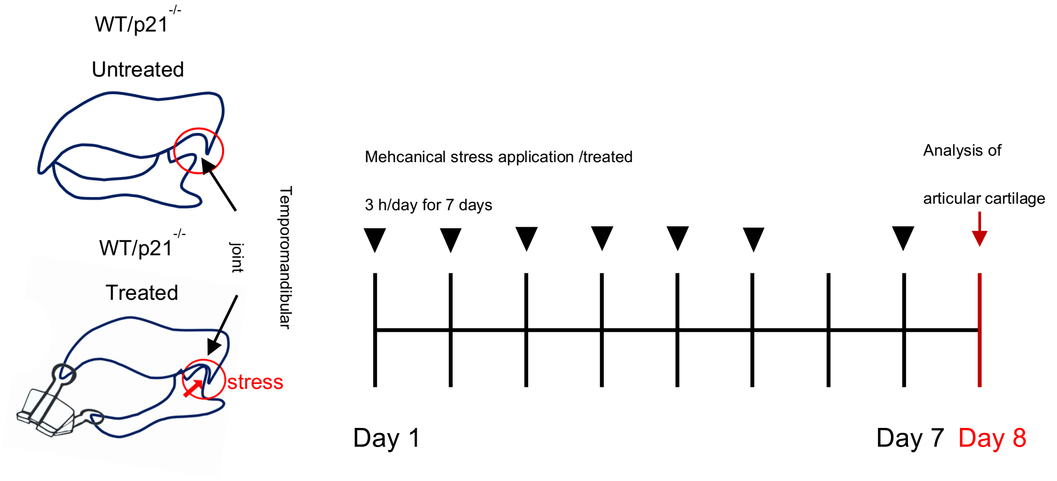

Supplemental Figure 1. The experiment and were randomly divided into two untreated and two treated groups: p21$^{-/-}$ and WT without mechanical stress for control groups; p21$^{-/-}$ and WT with mechanical stress for experimental groups (n = 5 for each group). In the treated groups, mechanical stress was applied to the TMJs by forced mouth opening for 3 hours/day for 7 days. This device kept the mandible in a maximal mouth opening position of 14 mm and delivered a force of 2 N on each TMJ.

Supplemental Figure 2

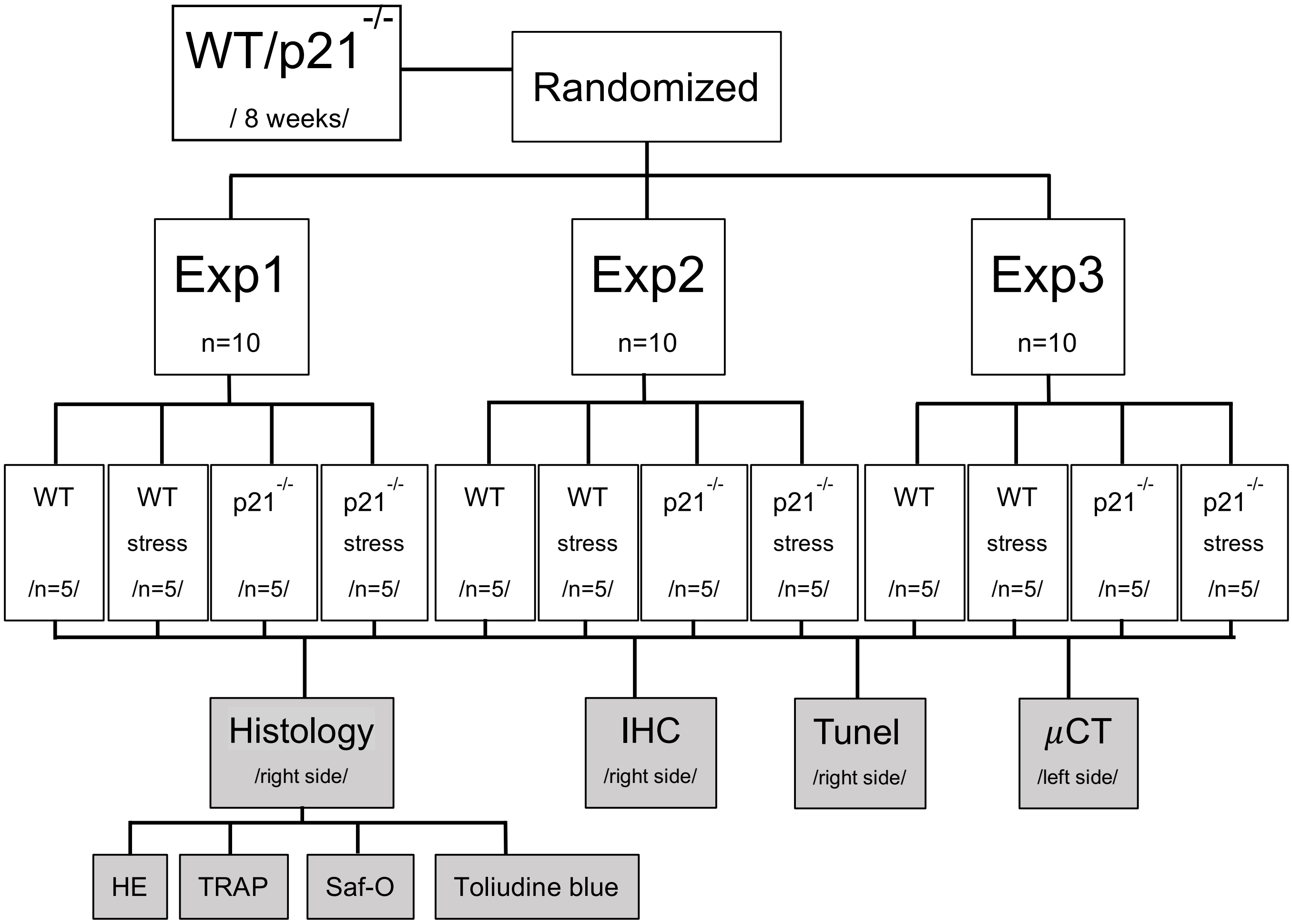

Supplemental Figure 2. Overview of experimental design (white box) and workflow (grey box).
